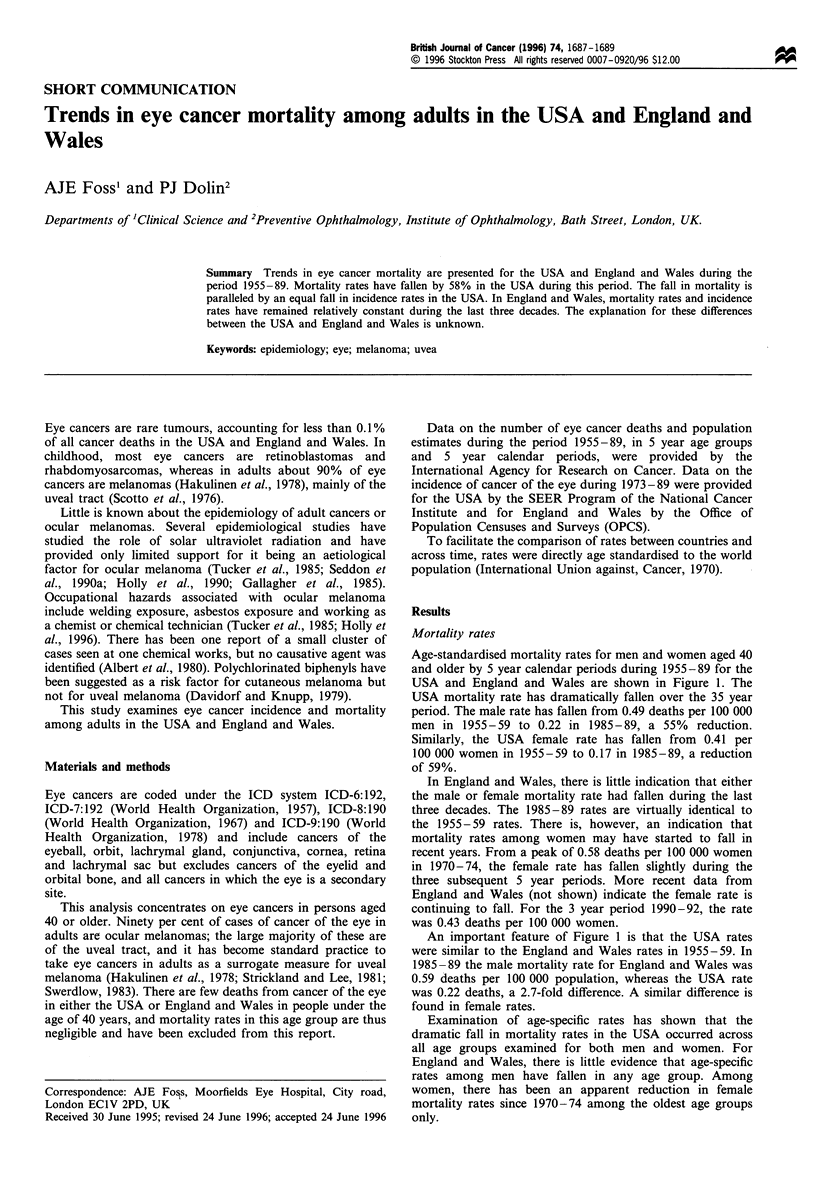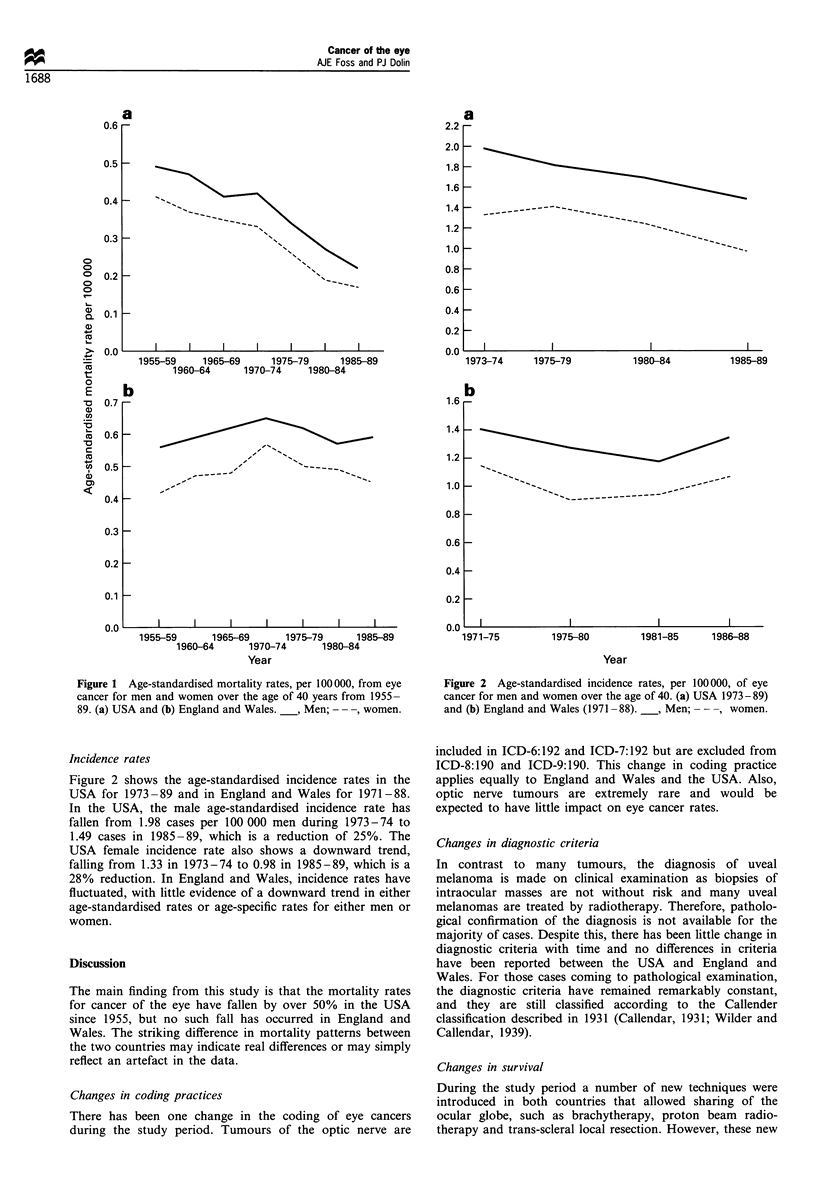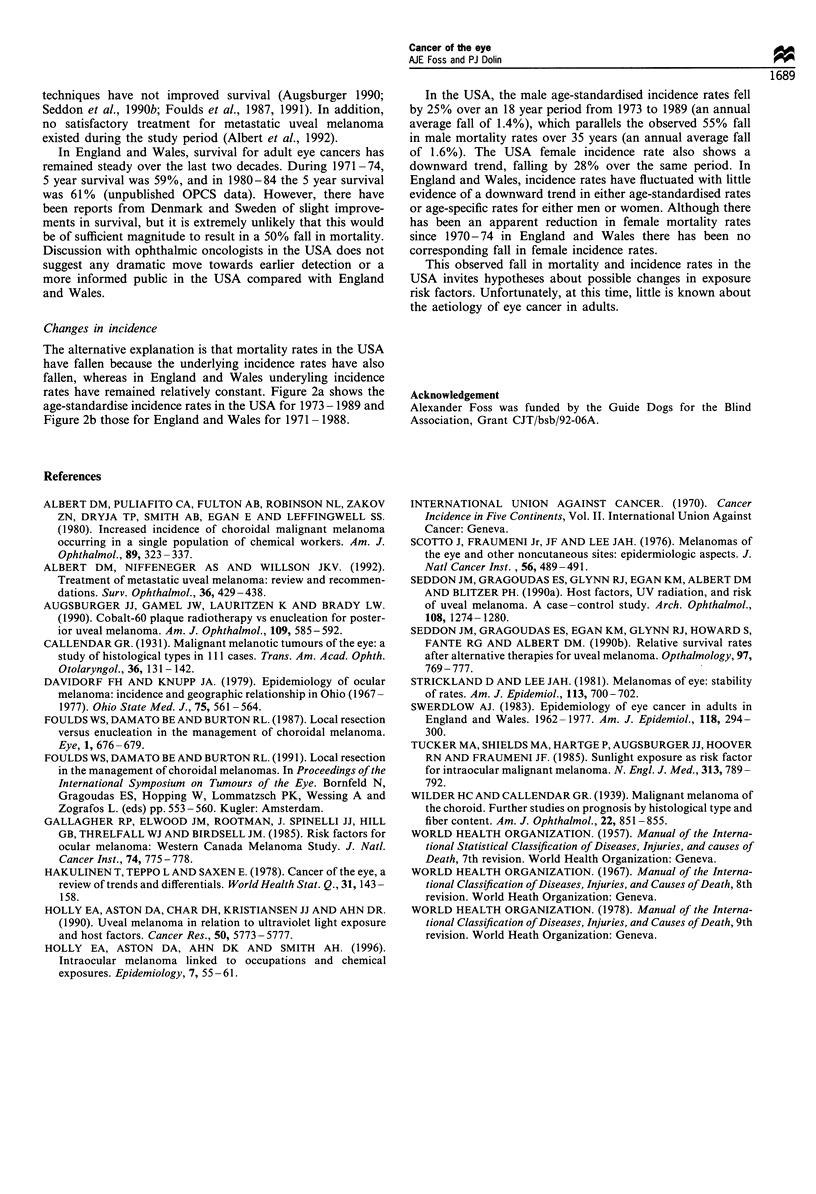# Trends in eye cancer mortality among adults in the USA and England and Wales.

**DOI:** 10.1038/bjc.1996.611

**Published:** 1996-11

**Authors:** A. J. Foss, P. J. Dolin

**Affiliations:** Department of Clinical Science, Institute of Ophthalmology, London, UK.

## Abstract

Trends in eye cancer mortality are presented for the USA and England and Wales during the period 1955-89. Mortality rates have fallen by 58% in the USA during this period. The fall in mortality is paralleled by an equal fall in incidence rates in the USA. In England and Wales, mortality rates and incidence rates have remained relatively constant during the last three decades. The explanation for these differences between the USA and England and Wales is unknown.


British Journal of Cancer (1996) 74, 1687-1689
© 1996 Stockton Press All rights reserved 0007-0920/96 $12.00

SHORT COMMUNICATION

### Trends in eye cancer mortality among adults in the USA and England and Wales

AJE Foss[1] and PJ Dolin[2]

*Departments of [1]Clinical Science and [2]Preventive Ophthalmology, Institute of Ophthalmology, Bath Street, London, UK.*

**Summary** Trends in eye cancer mortality are presented for the USA and England and Wales during the period 1955-89. Mortality rates have fallen by 58% in the USA during this period. The fall in mortality is paralleled by an equal fall in incidence rates in the USA. In England and Wales, mortality rates and incidence rates have remained relatively constant during the last three decades. The explanation for these differences between the USA and England and Wales is unknown.

**Keywords:** epidemiology; eye; melanoma; uvea

Eye cancers are rare tumours, accounting for less than 0.1% of all cancer deaths in the USA and England and Wales. In childhood, most eye cancers are retinoblastomas and rhabdomyosarcomas, whereas in adults about 90% of eye cancers are melanomas (Hakulinen *et al.*, 1978), mainly of the uveal tract (Scotto *et al.*, 1976).

Little is known about the epidemiology of adult cancers or ocular melanomas. Several epidemiological studies have studied the role of solar ultraviolet radiation and have provided only limited support for it being an aetiological factor for ocular melanoma (Tucker *et al.*, 1985; Seddon *et al.*, 1990a; Holly *et al.*, 1990; Gallagher *et al.*, 1985). Occupational hazards associated with ocular melanoma include welding exposure, asbestos exposure and working as a chemist or chemical technician (Tucker *et al.*, 1985; Holly *et al.*, 1996). There has been one report of a small cluster of cases seen at one chemical works, but no causative agent was identified (Albert *et al.*, 1980). Polychlorinated biphenyls have been suggested as a risk factor for cutaneous melanoma but not for uveal melanoma (Davidorf and Knupp, 1979).

This study examines eye cancer incidence and mortality among adults in the USA and England and Wales.

### Materials and methods

Eye cancers are coded under the ICD system ICD-6:192, ICD-7:192 (World Health Organization, 1957), ICD-8:190 (World Health Organization, 1967) and ICD-9:190 (World Health Organization, 1978) and include cancers of the eyeball, orbit, lachrymal gland, conjunctiva, cornea, retina and lachrymal sac but excludes cancers of the eyelid and orbital bone, and all cancers in which the eye is a secondary site.

This analysis concentrates on eye cancers in persons aged 40 or older. Ninety per cent of cases of cancer of the eye in adults are ocular melanomas; the large majority of these are of the uveal tract, and it has become standard practice to take eye cancers in adults as a surrogate measure for uveal melanoma (Hakulinen *et al.*, 1978; Strickland and Lee, 1981; Swerdlow, 1983). There are few deaths from cancer of the eye in either the USA or England and Wales in people under the age of 40 years, and mortality rates in this age group are thus negligible and have been excluded from this report.

Correspondence: AJE Foss, Moorfields Eye Hospital, City road, London EC1V 2PD, UK
Received 30 June 1995; revised 24 June 1996; accepted 24 June 1996

Data on the number of eye cancer deaths and population estimates during the period 1955-89, in 5 year age groups and 5 year calendar periods, were provided by the International Agency for Research on Cancer. Data on the incidence of cancer of the eye during 1973-89 were provided for the USA by the SEER Program of the National Cancer Institute and for England and Wales by the Office of Population Censuses and Surveys (OPCS).

To facilitate the comparison of rates between countries and across time, rates were directly age standardised to the world population (International Union against, Cancer, 1970).

### Results

### *Mortality rates*

Age-standardised mortality rates for men and women aged 40 and older by 5 year calendar periods during 1955-89 for the USA and England and Wales are shown in Figure 1. The USA mortality rate has dramatically fallen over the 35 year period. The male rate has fallen from 0.49 deaths per 100 000 men in 1955-59 to 0.22 in 1985-89, a 55% reduction. Similarly, the USA female rate has fallen from 0.41 per 100 000 women in 1955-59 to 0.17 in 1985-89, a reduction of 59%.

In England and Wales, there is little indication that either the male or female mortality rate had fallen during the last three decades. The 1985-89 rates are virtually identical to the 1955-59 rates. There is, however, an indication that mortality rates among women may have started to fall in recent years. From a peak of 0.58 deaths per 100 000 women in 1970-74, the female rate has fallen slightly during the three subsequent 5 year periods. More recent data from England and Wales (not shown) indicate the female rate is continuing to fall. For the 3 year period 1990-92, the rate was 0.43 deaths per 100 000 women.

An important feature of Figure 1 is that the USA rates were similar to the England and Wales rates in 1955-59. In 1985-89 the male mortality rate for England and Wales was 0.59 deaths per 100 000 population, whereas the USA rate was 0.22 deaths, a 2.7-fold difference. A similar difference is found in female rates.

Examination of age-specific rates has shown that the dramatic fall in mortality rates in the USA occurred across all age groups examined for both men and women. For England and Wales, there is little evidence that age-specific rates among men have fallen in any age group. Among women, there has been an apparent reduction in female mortality rates since 1970-74 among the oldest age groups only.

**Figure 1** Age-standardised mortality rates, per 100 000, from eye cancer for men and women over the age of 40 years from 1955–89. (**a**) USA and (**b**) England and Wales. ___, Men; – – –, women.

**Figure 2** Age-standardised incidence rates, per 100 000, of eye cancer for men and women over the age of 40. (**a**) USA 1973–89) and (**b**) England and Wales (1971–88). ___, Men; – – –, women.

*Incidence rates*

Figure 2 shows the age-standardised incidence rates in the USA for 1973–89 and in England and Wales for 1971–88. In the USA, the male age-standardised incidence rate has fallen from 1.98 cases per 100 000 men during 1973–74 to 1.49 cases in 1985–89, which is a reduction of 25%. The USA female incidence rate also shows a downward trend, falling from 1.33 in 1973–74 to 0.98 in 1985–89, which is a 28% reduction. In England and Wales, incidence rates have fluctuated, with little evidence of a downward trend in either age-standardised rates or age-specific rates for either men or women.

### Discussion

The main finding from this study is that the mortality rates for cancer of the eye have fallen by over 50% in the USA since 1955, but no such fall has occurred in England and Wales. The striking difference in mortality patterns between the two countries may indicate real differences or may simply reflect an artefact in the data.

*Changes in coding practices*

There has been one change in the coding of eye cancers during the study period. Tumours of the optic nerve are included in ICD-6:192 and ICD-7:192 but are excluded from ICD-8:190 and ICD-9:190. This change in coding practice applies equally to England and Wales and the USA. Also, optic nerve tumours are extremely rare and would be expected to have little impact on eye cancer rates.

*Changes in diagnostic criteria*

In contrast to many tumours, the diagnosis of uveal melanoma is made on clinical examination as biopsies of intraocular masses are not without risk and many uveal melanomas are treated by radiotherapy. Therefore, pathological confirmation of the diagnosis is not available for the majority of cases. Despite this, there has been little change in diagnostic criteria with time and no differences in criteria have been reported between the USA and England and Wales. For those cases coming to pathological examination, the diagnostic criteria have remained remarkably constant, and they are still classified according to the Callender classification described in 1931 (Callendar, 1931; Wilder and Callendar, 1939).

*Changes in survival*

During the study period a number of new techniques were introduced in both countries that allowed sharing of the ocular globe, such as brachytherapy, proton beam radiotherapy and trans-scleral local resection. However, these new techniques have not improved survival (Augsburger 1990; Seddon *et al.*, 1990*b*; Foulds *et al.*, 1987, 1991). In addition, no satisfactory treatment for metastatic uveal melanoma existed during the study period (Albert *et al.*, 1992).

In England and Wales, survival for adult eye cancers has remained steady over the last two decades. During 1971–74, 5 year survival was 59%, and in 1980–84 the 5 year survival was 61% (unpublished OPCS data). However, there have been reports from Denmark and Sweden of slight improvements in survival, but it is extremely unlikely that this would be of sufficient magnitude to result in a 50% fall in mortality. Discussion with ophthalmic oncologists in the USA does not suggest any dramatic move towards earlier detection or a more informed public in the USA compared with England and Wales.

*Changes in incidence*

The alternative explanation is that mortality rates in the USA have fallen because the underlying incidence rates have also fallen, whereas in England and Wales underyling incidence rates have remained relatively constant. Figure 2a shows the age-standardise incidence rates in the USA for 1973–1989 and Figure 2b those for England and Wales for 1971–1988.

In the USA, the male age-standardised incidence rates fell by 25% over an 18 year period from 1973 to 1989 (an annual average fall of 1.4%), which parallels the observed 55% fall in male mortality rates over 35 years (an annual average fall of 1.6%). The USA female incidence rate also shows a downward trend, falling by 28% over the same period. In England and Wales, incidence rates have fluctuated with little evidence of a downward trend in either age-standardised rates or age-specific rates for either men or women. Although there has been an apparent reduction in female mortality rates since 1970–74 in England and Wales there has been no corresponding fall in female incidence rates.

This observed fall in mortality and incidence rates in the USA invites hypotheses about possible changes in exposure risk factors. Unfortunately, at this time, little is known about the aetiology of eye cancer in adults.

**Acknowledgement**
Alexander Foss was funded by the Guide Dogs for the Blind Association, Grant CJT/bsb/92-06A.

### References

ALBERT DM, PULIAFITO CA, FULTON AB, ROBINSON NL, ZAKOV ZN, DRYJA TP, SMITH AB, EGAN E AND LEFFINGWELL SS. (1980). Increased incidence of choroidal malignant melanoma occurring in a single population of chemical workers. *Am. J. Ophthalmol.*, **89**, 323–337.

ALBERT DM, NIFFENEGER AS AND WILLSON JKV. (1992). Treatment of metastatic uveal melanoma: review and recommendations. *Surv. Ophthalmol.*, **36**, 429–438.

AUGSBURGER JJ, GAMEL JW, LAURITZEN K AND BRADY LW. (1990). Cobalt-60 plaque radiotherapy vs enucleation for posterior uveal melanoma. *Am. J. Ophthalmol.*, **109**, 585–592.

CALLENDAR GR. (1931). Malignant melanotic tumours of the eye: a study of histological types in 111 cases. *Trans. Am. Acad. Ophth. Otolaryngol.*, **36**, 131–142.

DAVIDORF FH AND KNUPP JA. (1979). Epidemiology of ocular melanoma: incidence and geographic relationship in Ohio (1967–1977). *Ohio State Med. J.*, **75**, 561–564.

FOULDS WS, DAMATO BE AND BURTON RL. (1987). Local resection versus enucleation in the management of choroidal melanoma. *Eye*, **1**, 676–679.

FOULDS WS, DAMATO BE AND BURTON RL. (1991). Local resection in the management of choroidal melanomas. In *Proceedings of the International Symposium on Tumours of the Eye*. Bornfeld N, Gragoudas ES, Hopping W, Lommatzsch PK, Wessing A and Zografos L. (eds) pp. 553–560. Kugler: Amsterdam.

GALLAGHER RP, ELWOOD JM, ROOTMAN, J. SPINELLI JJ, HILL GB, THRELFALL WJ AND BIRDSELL JM. (1985). Risk factors for ocular melanoma: Western Canada Melanoma Study. *J. Natl. Cancer Inst.*, **74**, 775–778.

HAKULINEN T, TEPPO L AND SAXEN E. (1978). Cancer of the eye, a review of trends and differentials. *World Health Stat. Q.*, **31**, 143–158.

HOLLY EA, ASTON DA, CHAR DH, KRISTIANSEN JJ AND AHN DR. (1990). Uveal melanoma in relation to ultraviolet light exposure and host factors. *Cancer Res.*, **50**, 5773–5777.

HOLLY EA, ASTON DA, AHN DK AND SMITH AH. (1996). Intraocular melanoma linked to occupations and chemical exposures. *Epidemiology*, **7**, 55–61.

INTERNATIONAL UNION AGAINST CANCER. (1970). *Cancer Incidence in Five Continents*, Vol. II. International Union Against Cancer: Geneva.

SCOTTO J, FRAUMENI Jr, JF AND LEE JAH. (1976). Melanomas of the eye and other noncutaneous sites: epidermiologic aspects. *J. Natl Cancer Inst.*, **56**, 489–491.

SEDDON JM, GRAGOUDAS ES, GLYNN RJ, EGAN KM, ALBERT DM AND BLITZER PH. (1990a). Host factors, UV radiation, and risk of uveal melanoma. A case–control study. *Arch. Ophthalmol.*, **108**, 1274–1280.

SEDDON JM, GRAGOUDAS ES, EGAN KM, GLYNN RJ, HOWARD S, FANTE RG AND ALBERT DM. (1990b). Relative survival rates after alternative therapies for uveal melanoma. *Opthalmology*, **97**, 769–777.

STRICKLAND D AND LEE JAH. (1981). Melanomas of eye: stability of rates. *Am. J. Epidemiol.*, **113**, 700–702.

SWERDLOW AJ. (1983). Epidemiology of eye cancer in adults in England and Wales. 1962–1977. *Am. J. Epidemiol.*, **118**, 294–300.

TUCKER MA, SHIELDS MA, HARTGE P, AUGSBURGER JJ, HOOVER RN AND FRAUMENI JF. (1985). Sunlight exposure as risk factor for intraocular malignant melanoma. *N. Engl. J. Med.*, **313**, 789–792.

WILDER HC AND CALLENDAR GR. (1939). Malignant melanoma of the choroid. Further studies on prognosis by histological type and fiber content. *Am. J. Ophthalmol.*, **22**, 851–855.

WORLD HEALTH ORGANIZATION. (1957). *Manual of the International Statistical Classification of Diseases, Injuries, and causes of Death*, 7th revision. World Health Organization: Geneva.

WORLD HEALTH ORGANIZATION. (1967). *Manual of the International Classification of Diseases, Injuries, and Causes of Death*, 8th revision. World Heath Organization: Geneva.

WORLD HEALTH ORGANIZATION. (1978). *Manual of the International Classification of Diseases, Injuries, and Causes of Death*, 9th revision. World Heath Organization: Geneva.